# Association of a Polygenic Risk Score With Osteoporosis in People Living With HIV: The Swiss HIV Cohort Study

**DOI:** 10.1093/infdis/jiad179

**Published:** 2023-05-24

**Authors:** Johannes M Schwenke, Christian W Thorball, Isabella C Schoepf, Lene Ryom, Barbara Hasse, Olivier Lamy, Alexandra Calmy, Gilles Wandeler, Catia Marzolini, Christian R Kahlert, Enos Bernasconi, Roger D Kouyos, Huldrych F Günthard, Bruno Ledergerber, Jacques Fellay, Felix Burkhalter, Philip E Tarr, for the Swiss HIV Cohort Study, I Abela, K Aebi-Popp, A Anagnostopoulos, M Battegay, E Bernasconi, D L Braun, H C Bucher, A Calmy, M Cavassini, A Ciuffi, G Dollenmaier, M Egger, L Elzi, J Fehr, J Fellay, H Furrer, C A Fux, H F Günthard, A Hachfeld, D Haerry, B Hasse, H H Hirsch, M Hoffmann, I Hösli, M Huber, D Jackson-Perry, C R Kahlert, L Kaiser, O Keiser, T Klimkait, R D Kouyos, H Kovari, K Kusejko, N Labhardt, K Leuzinger, B Martinez de Tejada, C Marzolini, K J Metzner, N Müller, J Nemeth, D Nicca, J Notter, P Paioni, G Pantaleo, M Perreau, A Rauch, L Salazar-Vizcaya, P Schmid, R Speck, M Stöckle, P Tarr, A Trkola, G Wandeler, M Weisser, S Yerly

**Affiliations:** University Department of Medicine and Infectious Diseases Service, Kantonsspital Baselland, University of Basel, Bruderholz; Precision Medicine Unit, Lausanne University Hospital, University of Lausanne; School of Life Sciences, Ecole Polytechnique Fédérale de Lausanne; University Department of Medicine and Infectious Diseases Service, Kantonsspital Baselland, University of Basel, Bruderholz; Department of Infectious Diseases, Bern University Hospital, University of Bern, Switzerland; CHIP, Centre of Excellence for Health, Immunity and Infections, Rigshospitalet, University of Copenhagen; Department of Infectious Diseases, Hvidovre University Hospital, Denmark; Department of Infectious Diseases and Hospital Epidemiology, University Hospital Zurich, University of Zurich; Bone Unit, Lausanne University Hospital, University of Lausanne; Division of Infectious Disease, Geneva University Hospital; Department of Infectious Diseases, Bern University Hospital, University of Bern, Switzerland; Division of Infectious Diseases and Hospital Epidemiology, University Hospital Basel; Division of Infectious Diseases, Kantonsspital St Gallen; Division of Infectious Diseases, Ospedale Regionale Lugano, University of Geneva and Università della Svizzera italiana, Lugano; Department of Infectious Diseases and Hospital Epidemiology, University Hospital Zurich, University of Zurich; Institute of Medical Virology, University of Zurich; Department of Infectious Diseases and Hospital Epidemiology, University Hospital Zurich, University of Zurich; Institute of Medical Virology, University of Zurich; Department of Infectious Diseases and Hospital Epidemiology, University Hospital Zurich, University of Zurich; Precision Medicine Unit, Lausanne University Hospital, University of Lausanne; School of Life Sciences, Ecole Polytechnique Fédérale de Lausanne; University Department of Nephrology and Dialysis, Kantonsspital Baselland, University of Basel, Bruderholz, Switzerland; University Department of Medicine and Infectious Diseases Service, Kantonsspital Baselland, University of Basel, Bruderholz

**Keywords:** aging, HIV infection, low bone mineral density, osteoporosis, polygenic risk score

## Abstract

**Background:**

Bone mineral density (BMD) loss may be accelerated in people with HIV (PLWH). It is unknown whether a polygenic risk score (PRS) is associated with low BMD in PLWH.

**Methods:**

Swiss HIV Cohort Study participants of self-reported European descent underwent ≥2 per-protocol dual x-ray absorptiometry (DXA) measurements ≥2 years apart (2011–2020). Univariable and multivariable odds ratios (ORs) for DXA-defined osteoporosis were based on traditional and HIV-related risk factors and a genome-wide PRS built from 9413 single-nucleotide polymorphisms associated with low BMD in the general population. Controls were free from osteoporosis/osteopenia on all DXA measurements.

**Results:**

We included 438 participants: 149 with osteoporosis and 289 controls (median age, 53 years; 82% male, 95% with suppressed HIV RNA). Participants with unfavorable osteoporosis PRS (top vs bottom quintile) had univariable and multivariable-adjusted osteoporosis ORs of 4.76 (95% CI, 2.34–9.67) and 4.13 (1.86–9.18), respectively. For comparison, hepatitis C seropositivity, 5-year tenofovir disoproxil fumarate exposure, and parent history of hip fracture yielded univariable osteoporosis ORs of 2.26 (1.37–3.74), 1.84 (1.40–2.43), and 1.54 (0.82–2.9).

**Conclusions:**

In PLWH in Switzerland, osteoporosis was independently associated with a BMD-associated PRS after adjustment for established risk factors, including exposure to tenofovir disoproxil fumarate.

## Teaser

An individual polygenic risk score was associated with osteoporosis in people living with HIV. The genetic effect was robust, as it persisted after multivariable adjustment for established traditional and HIV-related osteoporosis risk factors, including tenofovir disoproxil fumarate and boosted protease inhibitor exposure.

Bone health is a major long-term concern in people living with HIV (PLWH). Low-trauma fractures and low bone mineral density (BMD; ie, osteopenia and osteoporosis) are recorded more frequently in PLWH than in the general population [1, 2]. Increased osteoporosis susceptibility in PLWH has been attributed to a higher prevalence of traditional osteoporosis risk factors: low body weight, vitamin D deficiency, hepatitis C coinfection, smoking and other substance use, chronic inflammation, and toxicity from antiretroviral therapy (ART) agents such as tenofovir disoproxil fumarate (TDF) and boosted protease inhibitors (bPIs) [2–6].

BMD has a strong genetic component, with a heritability in the range of 50% to 90% [7–9]. Genome-wide association studies (GWASs) have now identified >500 single-nucleotide polymorphisms (SNPs) that are reliably associated with BMD in the general population [10, 11]. Furthermore, by combining the effect of the SNPs in these GWASs, it is possible to obtain a single measurement of the genetic risk conferred for the predicted outcome in the form of a polygenic risk score (PRS; reviewed by Torkamani et al [12]).

We previously reported on participants in the Swiss HIV Cohort Study (SHCS) regarding associations of GWAS-derived SNPs with dyslipidemia [13], diabetes mellitus [14], and low-trauma fractures [15], and we associated individual PRSs to chronic kidney disease [16], rapid progression of renal dysfunction [17], coronary artery disease events [18], and subclinical atherosclerosis [19]. The aim of the present study was to investigate whether an individual BMD-associated PRS is independently associated with osteoporosis in PLWH in Switzerland. We quantify the effect size of the PRS on osteoporosis risk in the context of multiple known clinical and HIV-related risk factors.

## METHODS

### Study Population

Participants included PLWH enrolled in the substudy “metabolism and aging” of the SHCS (www.shcs.ch) [20]. Inclusion criteria into the substudy were age ≥45 years; the ability to undergo neurocognitive testing in German, French, Italian, or English; and the ability to provide a fasting urine and plasma sample. Participants per protocol underwent ≥2 BMD measurements with a minimum scan interval ≥2 years. The study was approved by the local ethics committees. Participants provided written informed consent for substudy participation, including genetic testing.

### BMD Measurements

Lumbar spine and left hip BMD was measured by dual-energy x-ray absorptiometry (DXA) with Hologic or General Electric densitometers calibrated at regular intervals through standard phantoms. BMD measurements are given as grams per square centimeter (g/cm^2^). Osteoporosis was defined according to the guidelines of the European AIDS Clinical Society [21] as a T-score ≤ −2.5 SD of the lumbar spine (average at L1-L4) and/or left total hip on any DXA measurement or a Z-score ≤ −2 SD of the lumbar spine (average at L1-L4) and/or left total hip in premenopausal women and men aged < 50 years. Osteopenia was defined as a T-score ≤ −1.0 SD of the lumbar spine (average at L1-L4) and/or left total hip. Only per-protocol DXA measurements were included to minimize selection bias. We defined cases as participants with osteoporosis at any DXA scan. Controls had neither osteoporosis nor osteopenia—that is, all T-scores were > −1.0 SD, at all scans, to better separate the phenotypes. Similar to our previous work on PRSs associated with clinical (hard) coronary artery disease end points [18] and subclinical atherosclerosis [19], here we analyze only participants with DXA-defined osteoporosis; all participants with previous low-trauma fracture end points were excluded and will be analyzed separately.

### Nongenetic Osteoporosis Risk Factors

We defined all variables a priori, based on their osteoporosis association in the published literature, as previously reported [15]: age (per 10 years older), sex, menopausal status, smoking (current, past, never), body mass index (BMI) category (underweight, <18.5; normal, 18.5–24.9; overweight, 25–29.9; obese, >30), physical activity (>20 minutes of leisure activity per week vs less), HIV acquisition mode (heterosexual, men who have sex with men, injection drug use [IDU], other), diabetes mellitus (defined as plasma glucose >7.0 mmol/L [fasting] or >11.1 mmol/L [nonfasting] or receiving antidiabetic medication), dyslipidemia (defined as total cholesterol >6.2 mmol/L, HDL <1.0 mmol/L [males] or < 1.2 mmol/L [females], taking lipid-lowering therapy), parent history of hip fracture, treatment with corticosteroids (≥3 months), alcohol consumption (≥3 standard units daily vs less [15]), and hepatitis C virus (HCV) seropositivity. HIV-related risk factors included CD4 nadir (per 100 cells/μL higher and <50 cells/μL), HIV viremia at the time of DXA, maximal recorded HIV viremia, and cumulative exposure (per 5 years) to TDF and to bPIs such as ritonavir and cobicistat [2–6].

### Genotyping

As previously reported [18], DNA samples were obtained from peripheral blood mononuclear cells and genotyped with the Global Screening Array version 2.0 + MD (Illumina), if not already done in the setting of previous SHCS genetic studies. All quality control, filtering, and imputation steps prior to the merging of batches were performed separately for each batch of samples as described (Supplementary Methods). For the final merged data set used to calculate the PRS, only variants with a minor allele frequency >1% and missingness <10% were kept.

### Genome-wide PRSs

We calculated the PRS using PRSice (version 2.3.3) by directly applying the variant information from the BMD-associated PRS referred to as gSOS (genetically predicted heel quantitative ultrasound speed of sound) by Forgetta et al [22]. We downloaded information on included variants in the gSOS PRS and their weights from the PGS Catalog (PGS000657) [23]. As the gSOS PRS was validated with cohorts of predominantly European descent, our study population was likewise restricted to participants of European descent. Following *P*-value thresholding, 9413 SNPs were successfully matched and included in the gSOS PRS. Because of the prevalent concern that aging in PLWH may be accelerated and/or accentuated, we also assessed a PRS based on 4 SNPs that have been reliably associated with successful aging and longevity in the general population [24, 25], as we did for coronary event prediction in PLWH [18].

### Power Calculation

With a 2:1 ratio of cases and controls, 120 cases and 240 controls would be needed to detect an odds ratio (OR) >1.9 with a power of 0.8 and alpha of 0.05 [26].

### Statistical Analyses

We tested nongenetic and genetic factors associated with osteoporosis using univariable and multivariable standard logistic regression analyses. Age and an interaction term for sex with HIV acquisition mode were forced a priori into the multivariable model, whereas the other clinical covariates were entered if they had a *P* value < .05 in univariable analyses. Due to collinearity with sex and age, menopause association was analyzed only univariably. We stratified risk factors into quintiles to better visualize their potentially nonlinear associations with osteoporosis. Also, we combined all traditional and HIV-related risk factors into a single measure of “clinical” osteoporosis risk, by creating quintiles of the individually predicted osteoporosis event probabilities from the multivariable model with the clinical risk factors previously described. These clinical risk quintiles were then used to check for and visualize interactions with genetic risk factors. Model fit and interactions were analyzed with Akaike and bayesian information criteria and likelihood ratio tests. We used Stata/SE 17.0 (StataCorp).

### Sensitivity Analyses

We performed 4 sensitivity analyses to test the robustness of the association of the gSOS PRS with osteoporosis: first, we used the same case definition, with all other participants being controls (T-score > −2.5 or Z-score > −2 at both DXA scans); second, we used osteoporosis and/or osteopenia (T-score ≤ −1.0 or Z-score ≤ −2) as the case definition, with all other participants being controls (T-score > −1.0 at both DXA scans; Supplementary Figure 1). Additionally, because of potential collinearity between IDU and hepatitis C seropositivity, we conducted 2 additional separate multivariable analyses, each excluding 1 of these 2 covariates from the multivariable model.

## RESULTS

### Participants

**Figure 1.**
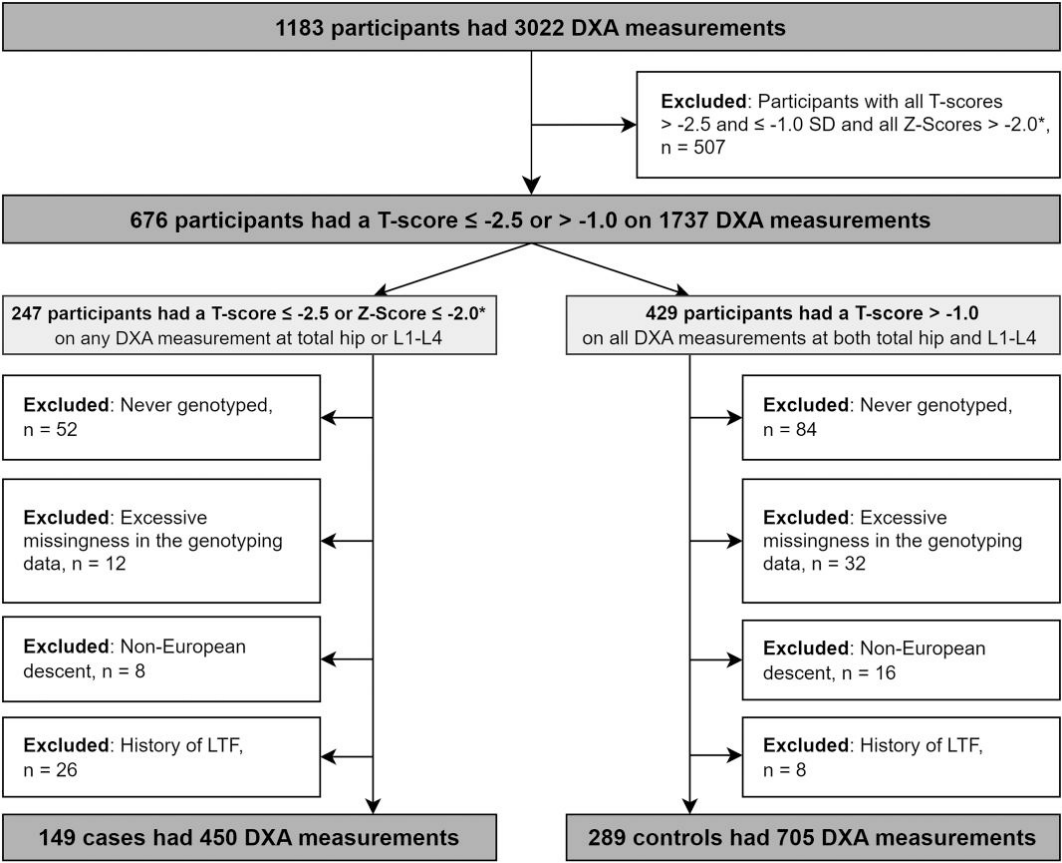
Participant disposition. *Z-scores were considered for premenopausal women and men aged <50 years at the time of DXA measurement. DXA, dual-energy x-ray absorptiometry; L1-L4, lumbar spine segments 1–4; LTF, low-trauma fracture.

Participant disposition is shown in Figure 1, and participant characteristics are shown in Table 1. The final study population consisted of 438 participants: 149 osteoporosis cases and 289 controls (334 participants with osteopenia were excluded from the primary analysis; Table 2). The median (IQR) date of DXAs was 11 June 2014 (21 January 2014–19 February 2015). Cases were older and more likely to be female, underweight, injection drug users, current smokers, and nondiabetic; their TDF and bPI exposure was also longer (Table 1).

**Table 1.** Characteristics of Cases and Controls and Osteoporosis Odds Ratio According to Clinical Risk Factors: Univariable and Multivariable Analysis Without Polygenic Risk Score

	Participants, Median (IQR) or No. (%)			Analysis, OR (95% CI); *P* Value	
	All (N = 438)	Cases (n = 149)	Controls (n = 289)	Univariable	Multivariable
T-score					
Lumbar spine, L1-L4	−0.5 (−2.5 to 0.4)	−2.8 (−3.1 to −2.5)	0.0 (−0.5 to 0.8)	…	…
Left total hip	−0.5 (−1.3 to 0.2)	−1.9 (−2.4 to −1.3)	−0.1 (−0.5 to 0.4)	…	…
Male sex	360 (82.2)	119 (79.9)	241 (83.4)	1.27 (.76–2.10) ^a^; .362	… ^b^
Age, y	53 (50–59)	55 (51–61)	52 (49–58)	1.36 (1.04–1.77) ^c^; .024	1.40 (1.02–1.92) ^c^; .037
Menopause^d^	46 (59.0)	25 (83.3)	21 (43.8)	6.42 (2.10–19.64); .001	…^e^
Body mass index					
Underweight, <18.5	19 (4.3)	18 (12.1)	1 (0.4)	21.12 (2.77–160.87); .003	20.97 (2.55–172.25); .005
Normal, 18.5–24.9	226 (51.6)	104 (69.8)	122 (42.2)	1 [Reference]	1 [Reference]
Overweight, 25–29.9	146 (33.3)	22 (14.8)	124 (42.9)	0.21 (.12–.35); < .001	0.20 (.12–.36); < .001
Obese, >30	47 (10.7)	5 (3.4)	42 (14.5)	0.14 (.05–.37); < .001	0.16 (.06–.44); < .001
Physical activity, >20 min ≥1/wk	230 (52.5)	67 (45.0)	163 (56.4)	0.63 (.42–.94); .024	0.67 (.42–1.07); .091
HIV acquisition mode					
Heterosexual	133 (30.4)	42 (28.2)	91 (31.5)		
Male				1.04 (.59–1.81); .902	0.88 (.45–1.72); .712
Female				0.95 (.51–1.76); .864	0.62 (.29–1.32); .215
MSM	240 (54.8)	76 (51.0)	164 (56.8)	1 [Reference]	1 [Reference]
IDU	50 (11.4)	29 (19.5)	21 (7.3)		
Male				2.41 (1.19–4.90); .015	1.45 (.54–3.90); .465
Female				5.39 (1.64–17.75); .006	2.72 (.66–11.29); .167
Other	15 (3.4)	2 (1.3)	13 (4.5)	0.33 (.07–1.51); .153	0.41 (.08–2.01); .269
Smoking					
Current	160 (36.5)	61 (40.9)	99 (34.3)	1.39 (.86–2.23); .180	…
Past	135 (30.8)	44 (29.5)	91 (31.5)	1.09 (.66–1.80); .744	…
Never	143 (32.7)	44 (29.5)	99 (34.3)	1 [Reference]	…
Alcohol consumption					
None/mild	246 (56.2)	86 (57.7)	160 (55.4)	1 [Reference]	…
Moderate/heavy	192 (43.8)	63 (42.3)	129 (44.6)	0.91 (.61–1.35); .638	…
Diabetes mellitus	30 (6.9)	5 (3.4)	25 (8.7)	0.37 (.14–.98); .045	0.48 (.14–1.58); .225
Dyslipidemia	197 (45.0)	64 (43.0)	133 (46.0)	0.88 (.59–1.32); .541	…
Lipid-lowering therapy	64 (14.6)	23 (15.4)	41 (14.2)	1.10 (.63–1.92); .726	…
Corticotherapy > 3 mo	23 (5.3)	6 (4.0)	17 (5.9)	0.67 (.26–1.74); .412	…
Hepatitis C seropositivity	76 (17.4)	38 (25.5)	38 (13.2)	2.26 (1.37–3.74); .001	0.98 (.44–2.15); .951
Parent hip fracture	44 (10.1)	19 (12.8)	25 (8.7)	1.54 (.82–2.90); .179	…
Exposure, y					
Tenofovir disoproxil fumarate	4.4 (1.0–7.5)	5.8 (2.6–8.7)	3.5 (0.3–6.9)	1.84 (1.40–2.43) ^f^; < .001	1.87 (1.34–2.60) ^f^; < .001
Boosted protease inhibitor	2 (0.0–7.8)	5 (0.0–9.3)	1.3 (0.0–5.9)	1.44 (1.18–1.76) ^f^; < .001	1.08 (.84–1.40) ^f^; .532
CD4					
Nadir, cells/μL	199 (99–286)	171 (90–260)	210 (104–304)	0.84 (.73–.97); .020	1.04 (.87–1.23); .672
Nadir, <50 cells/μL	55 (12.6)	19 (12.8)	36 (12.5)	1.03 (.57–1.86); .930	…
Cells/μL	644 (488–852)	611 (463–812)	652 (507–861)	0.97 (.91–1.04); .370	…
HIV RNA					
Undetectable, <50 copies/mL	415 (94.8)	141 (94.6)	274 (94.8)	0.96 (.40–2.33); .937	…
Maximal, copies/mL, log	5.2 (4.6–5.7)	5.1 (4.5–5.6)	5.2 (4.7–5.7)	0.87 (.71–1.07); .182	…

All data apply to the time point of the first dual x-ray absorptiometry scan.

Abbreviations: IDU, injection drug use; MSM, men who have sex with men; OR, odds ratio.

Female sex.

Sex is considered separately for injection drug users and heterosexual participants under “HIV acquisition mode.”

Per 10 years older.

Menopause status was considered only for female participants (n = 78).

Due to collinearity with sex and age, menopause association was analyzed only univariably.

Per 5-year exposure.

**Table 2.** Multivariable-Adjusted Odds Ratios for Osteoporosis According to gSOS PRS Quintile

	Analysis, OR (95% CI) ^a^; *P* Value		
	Primary ^b^	Sensitivity: Control ^c^	Sensitivity: Case ^d^
Normal BMD ^e^	Controls (n = 289)	Controls (n = 623)	Controls (n = 289)
Osteopenia ^f^	Excluded (n = 334)		Cases (n = 483)
Osteoporosis ^g^	Cases (n = 149)	Cases (n = 149)	
gSOS PRS quintile vs first			
Second	2.53 (1.11–5.75); 0.027	1.63 (.81–3.26); .171	1.26 (.78–2.04); .349
Third	2.88 (1.27–6.55); .011	2.41 (1.23–4.73); .010	1.57 (.97–2.55); .066
Fourth	2.70 (1.20–6.03); .016	2.05 (1.05–3.99); .035	1.36 (.83–2.22); .217
Fifth	4.13 (1.86–9.18); < .001	2.34 (1.20–4.57); .013	2.30 (1.37–3.88); .002

Abbreviations: BMD, bone mineral density; gSOS, genetically predicted heel quantitative ultrasound speed of sound; OR, odds ratio; PRS, polygenic risk score.

All odds ratios are adjusted for traditional and HIV-related risk factors, including antiretrovirals.

Primary analysis: cases are defined as any T-score ≤ −2.5 or Z-score ≤ −2 in premenopausal women or men aged <50 years and with controls defined as all T-scores > −1.0.

Sensitivity analysis with controls defined as all T-scores > −2.5.

Sensitivity analysis with cases defined as any T-score ≤ −1.0 or Z-score ≤ −2 in premenopausal women or men aged <50 years.

T-score > −1.0.

T-score ≤ −1.0 and > −2.5.

T-score ≤ −2.5 or Z-score ≤ −2 in premenopausal women or men aged <50 years.

### Probability of Osteoporosis

#### Univariable Analysis

**Figure 2.**
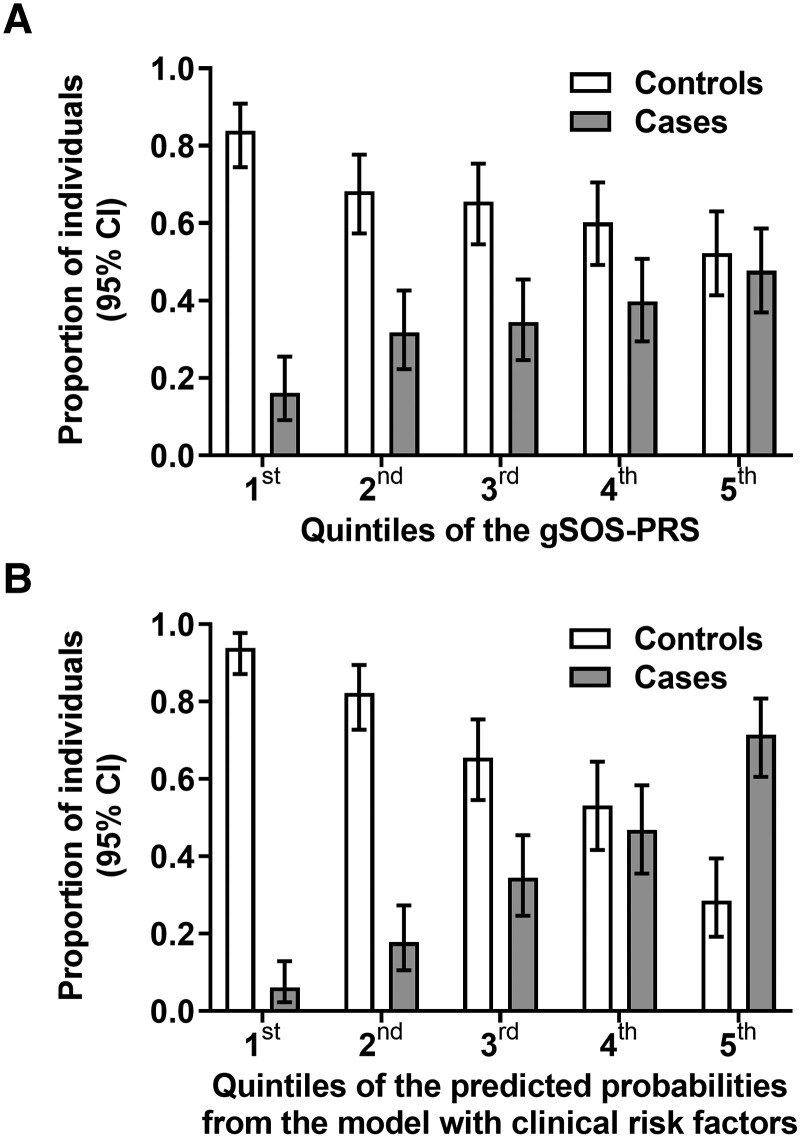
Distribution of PRSs and clinical risk factors in 289 controls without osteopenia or osteoporosis and 149 cases with osteoporosis. We divided study participants into cases and controls and into 5 quintiles according to their individual gSOS PRSs and clinical risk factors combined. Proportion and 95% CI of cases and controls are presented per quintile. *A*, Distribution of osteoporosis cases and controls according to quintiles of the gSOS PRS. There were 14 (16.1%) cases vs 73 (83.9%) controls in the first quintile (most favorable), 28 (31.8%) vs 60 (68.2%) in the second, 30 (34.5%) vs 57 (65.5%) in the third, 35 (39.8%) vs 53 (60.2%) in the fourth, 42 (47.7%) vs 46 (52.3%) in the fifth (least favorable). *B*, Distribution of osteoporosis cases and controls according to quintiles of clinical risk. There were 6 (6.1%) cases vs 92 (93.9%) controls in the first quintile (most favorable), 16 (17.8%) vs 74 (82.2%) in the second, 30 (34.5%) vs 57 (65.5%) in the third, 37 (46.8%) vs 42 (53.2%) in the fourth, 60 (71.4%) vs 24 (28.6%) in the fifth (least favorable). gSOS, genetically predicted heel quantitative ultrasound speed of sound; PRS, polygenic risk score.

**Figure 3.**
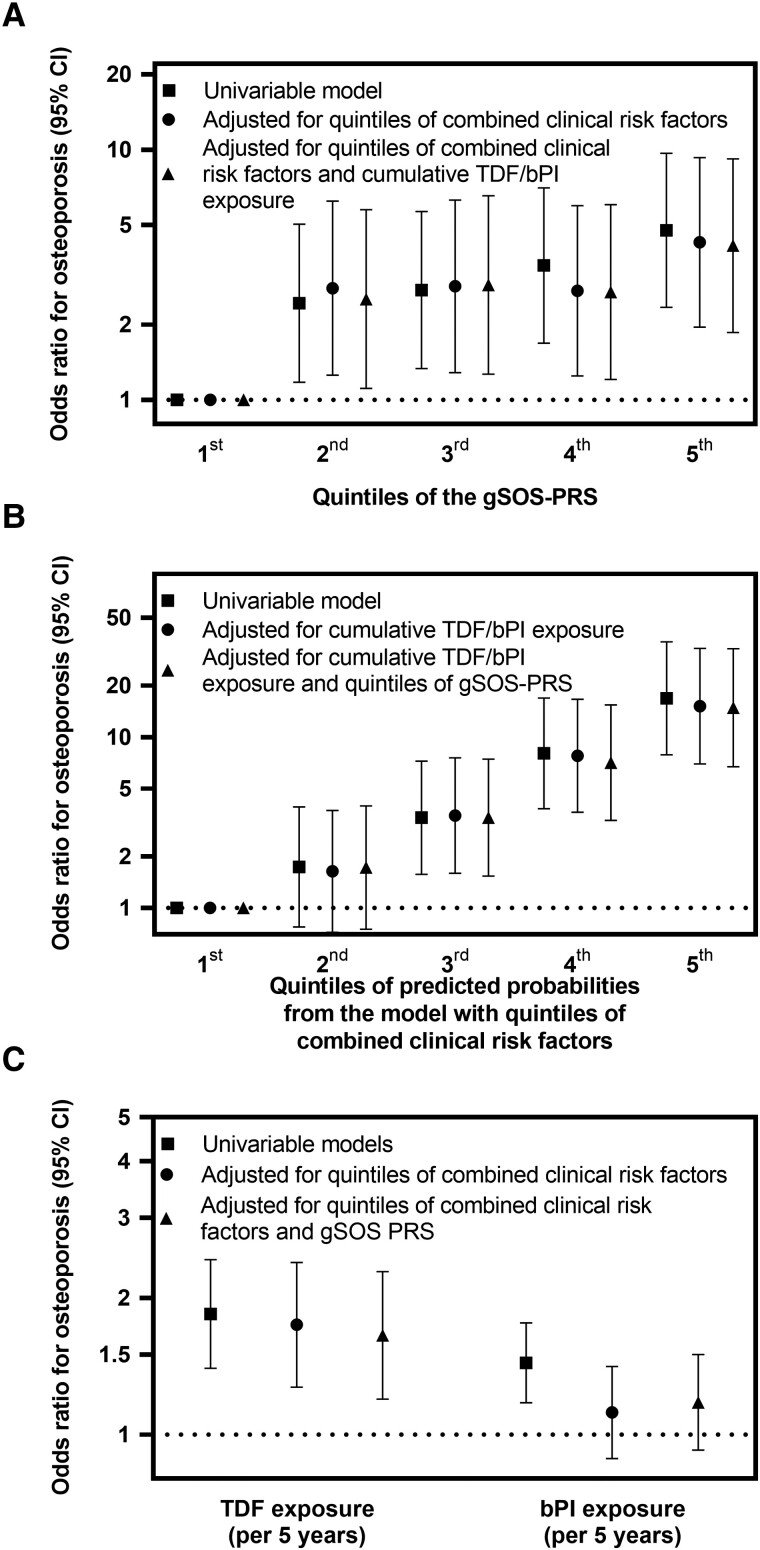
Odds ratios for osteoporosis according to the gSOS PRS, clinical risk factors, and TDF and bPI. Univariable and multivariable odds ratios and 95% CIs for osteoporosis according to (*A*) quintiles of the gSOS PRS, (*B*) quintiles of clinical risk factors, and (*C*) cumulative exposure to TDF and bPI. Results show simple logistic regression of associations with osteoporosis for 149 osteoporosis cases and 289 osteoporosis- and osteopenia-free controls. All odds ratios and 95% CIs are tabulated in Supplementary Table 2. bPI, boosted protease inhibitor; gSOS, genetically predicted heel quantitative ultrasound speed of sound; PRS, polygenic risk score; TDF, tenofovir disoproxil fumarate.

Osteoporosis probability was significantly associated with the gSOS PRS (*P* < .001; Figure 2*A*) but not with parent history of hip fracture (*P* = .179) or the longevity PRS (*P* = .61; Supplementary Table 1). When compared with the first gSOS PRS quintile (most favorable), participants in the second, third, fourth, and fifth (most unfavorable) had increased risk of osteoporosis, with ORs of 2.43 (95% CI, 1.18–5.03), 2.74 (1.33–5.65), 3.44 (1.69–7.03), and 4.76 (2.34–9.67), respectively (Figure 3*A*, Supplementary Table 2). For comparison, in univariable osteoporosis, the OR was 1.84 (95% CI, 1.40–2.43) for 5-year TDF exposure, 1.44 (1.18–1.76) for 5-year bPI exposure, and 2.26 (1.37–3.74) for hepatitis C seropositivity (Table 1). Osteoporosis probability was significantly associated with combined clinical risk factors (*P* < .001; Figures 2*B* and 3*B*, Supplementary Table 2). The gSOS PRS was not associated with any clinical covariate except BMI (Spearman ρ = −0.10, *P* = .005).

#### Multivariable Analysis

Participants in the second, third, fourth, and fifth quintiles of the gSOS PRS had increased risk of osteoporosis (vs the first quintile), with ORs of 2.53 (95% CI, 1.11–5.75), 2.88 (1.27–6.55), 2.70 (1.20–6.03), and 4.13 (1.86–9.18), respectively (Table 2, Figure 3*A*). For comparison, osteoporosis probability remained associated with cumulative TDF exposure (OR per 5 years, 1.65; 95% CI, 1.20–2.29) but not cumulative bPI exposure (OR per 5 years, 1.18; .92–1.50) or hepatitis C seropositivity (OR, 0.98; .44–2.15; Supplementary Table 2, Figure 3*C*). Osteoporosis probability remained significantly associated with combined clinical risk factors (Figure 3*B*).

### Sensitivity Analyses

#### Alternative Control Definition Including Osteopenia

When we defined controls as all participants without osteoporosis, the gSOS PRS remained independently associated with osteoporosis, but the effect size was smaller. This was expected, since participants with osteopenia were included among the controls. For example, in multivariable analysis, participants in the fifth gSOS PRS quintile (vs the first) had an increased osteoporosis risk, with an OR of 2.34 (95% CI, 1.20–4.57; Table 2, Supplementary Figure 1, Supplementary Table 3).

#### Alternative Case Definition Including Osteoporosis and Osteopenia

When we defined cases as all participants with a T-score ≤ −1.0 at any DXA scan and controls as all other participants, the gSOS PRS remained independently associated with osteoporosis, but the effect size was smaller and statistically significant in only the fifth quintile. For example, in multivariable analysis, participants in the fifth gSOS PRS quintile (vs the first) had an increased osteoporosis risk, with an OR of 2.30 (1.37–3.88; Table 2, Supplementary Figure 2, Supplementary Table 4).

#### Inclusion of IDU or Hepatitis C Seropositivity in the Final Model

When we included only IDU (but not hepatitis C) in the model, results remained similar: participants in the fifth gSOS quintile had osteoporosis with an OR of 3.27 (95% CI, 1.46–7.32) as compared with the first gSOS quintile (Supplementary Table 5). Furthermore, in multivariable analysis including only hepatitis C (but not IDU) in the model, results remained similar: participants in the fifth gSOS PRS quintile had osteoporosis with an OR of 3.43 (95% CI, 1.54–7.63) as compared with the first gSOS PRS quintile (Supplementary Table 6).

## DISCUSSION

Here we analyzed a PRS for osteoporosis in PLWH in the context of traditional and HIV-related factors, including osteoporosis-associated antiretrovirals. Our genetic study has 4 main findings. First, when an individual PRS based on 9413 BMD-associated SNPs was applied, an unfavorable genetic background independently increased osteoporosis risk approximately 4-fold. Second, this effect of an unfavorable genetic background appears clinically relevant because it was larger than the effect of well-established osteoporosis risk factors, such as TDF exposure for 5 years. Third, an unfavorable genetic background also increased the risk of the combined end point of osteoporosis or osteopenia. As expected (because of the less stringent separation of cases and controls), the genetic effect size was smaller and seen only in participants with the most unfavorable genetic background; that is, participants in the top PRS quintile had a 2.3-fold increase in osteopenia/osteoporosis risk. Fourth, the association of the gSOS PRS and osteoporosis was not affected by parent history of hip fracture, as we were unable to document any association of parent history with osteoporosis. We found no evidence for any association of the longevity PRS with osteoporosis, in contrast to our recent study of coronary artery disease events in PLWH [18].

Our results may inform future research, as it is likely that PRS for various aging-associated end points (cardiovascular, diabetes, kidney, bone, etc) will enter routine medical care in the next 10 to 15 years. Knowledge of an unfavorable osteoporosis PRS may provide an early opportunity to obtain DXA and suggest to HIV clinicians to pay even greater attention to the management of clinical risk factors, such as smoking cessation, optimal calcium and vitamin D intake, optimization of the ART regimen, and promotion of a physically active lifestyle. Nonetheless, it was beyond the scope of our study to investigate the clinical value of genetic testing in PLWH for osteoporosis prediction (this would require prospective studies).

Our results, documenting an independent association of an unfavorable PRS with osteoporosis in PLWH, appear robust because the association persisted after adjustment for multiple established risk factors, including potentially osteoporosis-associated antiretrovirals, and in multiple sensitivity analyses. Additional strengths of the study are the exploitation of prospectively recorded information in participants of the well-established SHCS, allowing us to quantify and compare the effects of all relevant osteoporosis risk factors. We restricted the genetic variants analyzed to a PRS that has been extensively validated in the general population [22, 27], and we applied rigorous quality control to the genetic data, correcting for residual population stratification and excluding population outliers. Importantly, we excluded patients with fractures (these will be analyzed separately) and included only DXA measurements obtained per protocol [20], thereby minimizing selection bias.

We confirm the strong inverse association of BMD with BMI, consistent with studies in the general population [28–30] and PLWH [31, 32]. The association of the gSOS PRS with BMI was unexpected because in larger data sets from the general population [27], no such association was found. The negative direction of the effect (Spearman ρ = −0.10) in our data set is biologically plausible: a higher gSOS PRS (indicating increased osteoporosis risk) was associated a lower BMI, a well-established osteoporosis risk factor [28–30]. This might suggest that the osteoporosis risk conveyed by a high gSOS PRS is partially mediated by decreased bodyweight, but this association needs to be confirmed in other patient populations before considering it a true finding.

In our study, HCV seropositivity was not associated with osteoporosis after adjusting for potential confounders. The literature on the effects of HCV on BMD are mixed [33–35]. HCV seropositivity appears to be more robustly predictive of fracture risk in PLWH [36]; that is, HCV might increase fracture risk through other pathways than by decreasing BMD [36].

Our study has limitations. We included only participants of European descent because the gSOS PRS was developed in cohorts of predominantly European descent. The current Eurocentric nature of most GWASs and PRSs may exacerbate health disparities, and diversifying efforts are urgently needed [37]. Because our population was 82% male and relatively young, results should cautiously be applied to female and elderly PLWH. We were unable to analyze potentially osteoporosis-related non-ART medication, such as anticonvulsants, hormone replacement therapy, and bisphosphonates. The SHCS routinely started collecting these data in 2015 (ie, after the majority of DXA measurements were obtained in our study) and does not routinely capture data on male hypogonadism. Causal relationships may be revealed and pathogenic insights afforded by detailed genetic pathway analyses, as applied by the methods of mendelian randomization [38]. Based on a limited sample size, our study was not powered for these kinds of genetic analyses. While the effect size of PRS on osteoporosis risk appears larger than the effect size of potentially osteoporosis-associated ART, the effects of TDF/bPI exposure on bone health may become apparent only with accumulating years of exposure [4], and HIV clinicians have increasingly been deprescribing TDF/bPI for bone, kidney, and other health reasons. Furthermore, a parent’s history of hip fracture was recorded in relatively few participants (10%); that is, our power to detect such an association might have been limited. Finally, even though our study population had few specific inclusion criteria, our results may not be broadly applicable to all PLWH, as shown by the observation that some expected variables did not associate with osteoporosis, such as smoking, alcohol, and CD4 nadir.

In conclusion, osteoporosis is of considerable concern in PLWH—for whom osteoporosis risk factors and the cumulative effects of certain ART agents may be prevalent and aging may be accentuated or even accelerated [39, 40]. In our study of PLWH in Switzerland, an unfavorable genetic background, as captured by an individual PRS, independently increased osteoporosis risk 4-fold when adjusted for multiple clinical risk factors, including ART.

## Supplementary Data

Supplementary materials are available at *The Journal of Infectious Diseases* online. Consisting of data provided by the authors to benefit the reader, the posted materials are not copyedited and are the sole responsibility of the authors, so questions or comments should be addressed to the corresponding author.

## Supplementary Material

jiad179_Supplementary_Data

## Notes

***Author contributions*.** Study design: J. M. S., I. C. S., C. W. T., B. L., R. D. K., H. F. G., J. F., F. B., P. E. T. Data management, participant selection: B. L. Data acquisition: B. H., O. L., A. C., G. W., C. M., C. R. K., E. B., P. E. T. Data analysis: J. M. S., C. W. T., I. C. S., L. R., B. L., J. F., F. B., P. E. T. Drafting of the manuscript: J. M. S., P. E. T. Critical review and revision of the manuscript: All authors.

***Acknowledgments*.** The authors acknowledge the effort and commitment of investigators, study nurses, laboratory personnel, and participants.

***Financial support.*** This study has been financed within the framework of the SHCS, supported by the Swiss National Science Foundation (grant #201369), SHCS project #859, and the SHCS Research Foundation. The data are gathered by the 5 Swiss university hospitals, 2 cantonal hospitals, 15 affiliated hospitals, and 36 private physicians (listed in http://www.shcs.ch/180-health-care-providers). Funding to pay the Open Access publication charges for this article was provided by University of Basel.

***Members of the Swiss HIV Cohort Study.*** I. Abela, K. Aebi-Popp, A. Anagnostopoulos, M. Battegay, E. Bernasconi, D. L. Braun, H. C. Bucher, A. Calmy, M. Cavassini, A. Ciuffi, G. Dollenmaier, M. Egger, L. Elzi, J. Fehr, J. Fellay, H. Furrer, C. A. Fux, H. F. Günthard (president of the SHCS), A. Hachfeld, D. Haerry (deputy of Positive Council), B. Hasse, H. H. Hirsch, M. Hoffmann, I. Hösli, M. Huber, D. Jackson-Perry (patient representative), C. R. Kahlert (chairman of the Mother and Child Substudy), L. Kaiser, O. Keiser, T. Klimkait, R. D. Kouyos, H. Kovari, K. Kusejko (head of Data Centre), N. Labhardt, K. Leuzinger, B. Martinez de Tejada, C. Marzolini, K. J. Metzner, N. Müller, J. Nemeth, D. Nicca, J. Notter, P. Paioni, G. Pantaleo, M. Perreau, A. Rauch (chairman of the Scientific Board), L. Salazar-Vizcaya, P. Schmid, R. Speck, M. Stöckle (chairman of the Clinical and Laboratory Committee), P. Tarr, A. Trkola, G. Wandeler, M. Weisser, S. Yerly.
