# Supplementary material for: Association of a Polygenic Risk Score With Osteoporosis in People Living With HIV: The Swiss HIV Cohort Study
Source: J Infect Dis. 2023 May 24;228(6):742–50. doi: 10.1093/infdis/jiad179 (PMC10503954; doi:10.1093/infdis/jiad179)
Supplement: jiad179_Supplementary_Data [file jiad179_supplementary_data.docx]

SUPPLEMENTARY MATERIAL (online only)

**Association of a Polygenic Risk Score with Osteoporosis in Persons Living with HIV: The Swiss HIV Cohort Study**

Johannes M. Schwenke, Christian W. Thorball et al.

# Supplementary Methods

**Genotyping, Quality Control.** For each genotyping batch, samples and SNPs were removed if having an excessive genotype missingness (>10%) or if the minor allele frequency deviated more than 20% from that of the 1000 Genomes Project Phase 3 EUR reference panel. Missing genotypes were imputed using positional Burrows-Wheeler transformation (PBWT) [1], at the Sanger Imputation Service [2], using the 1000 Genomes Project Phase 3 panel as reference. Phasing was performed using EAGLE2 [3]. Only high-quality SNPs with an imputation information score (INFO > 0.8) were retained following the imputation, after which the genotyping batches were combined. Principal components and population structure was calculated with EIGENSTRAT (v6.1.4) [4], together with the HapMap3 reference panel [5]. For the subsequent genetic risk score, only individuals clustering with the European HapMap3 samples were included. The cohort was furthermore screened with KING (v2.1.3) [6] to ensure that no cryptic related or duplicate samples were included. Lastly, samples and SNPs with excessive missingness (above 10%), low minor allele frequency (below 1%) or excessive deviation from Hardy-Weinberg Equilibrium (P_HWE_ < 1e-6) were removed prior to calculating the genetic risk scores.

## Supplementary Figure 1: Flowchart of the Inclusion into Sensitivity Analyses 1: Same Case Definition as in the Primary Analysis and a Control Definition of T-score > -2.5 at all DXA Scans.

**
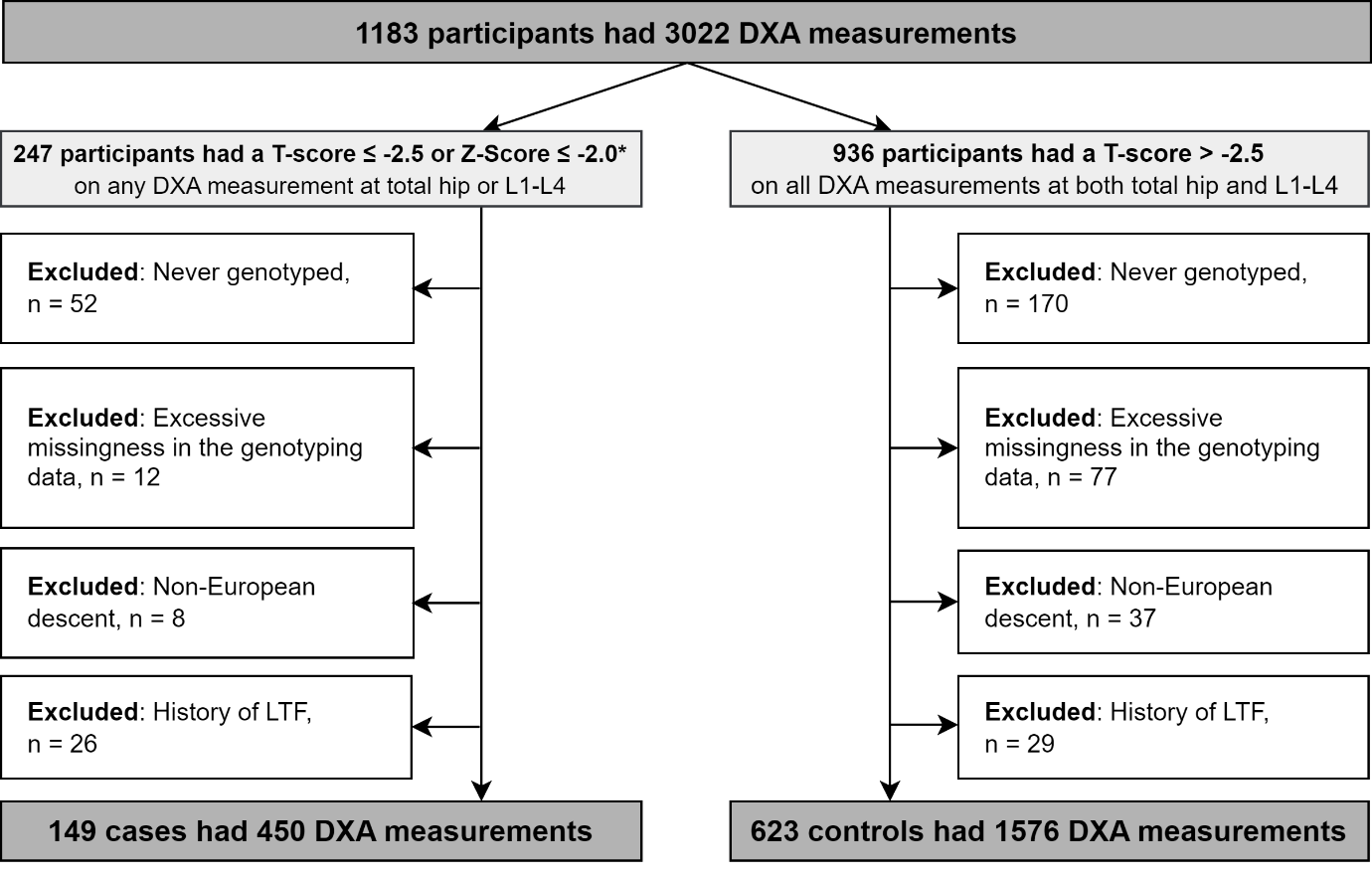
**

***** Z-Scores were considered for premenopausal women and men <50 years at the time of DXA-measurement.

**Abbreviations**: DXA, dual-energy X-ray absorptiometry; L1-L4, lumbar spine segments 1-4; LTF, low trauma fracture.

## Supplementary Figure 2: Flowchart of the Inclusion into Sensitivity Analysis 2: T-Score < -1.0 or Z-score < -2 in premenopausal women or men < 50 years as Case Definition, Participants with a T-Score > -1.0 on all DXA Scans as Controls.

**
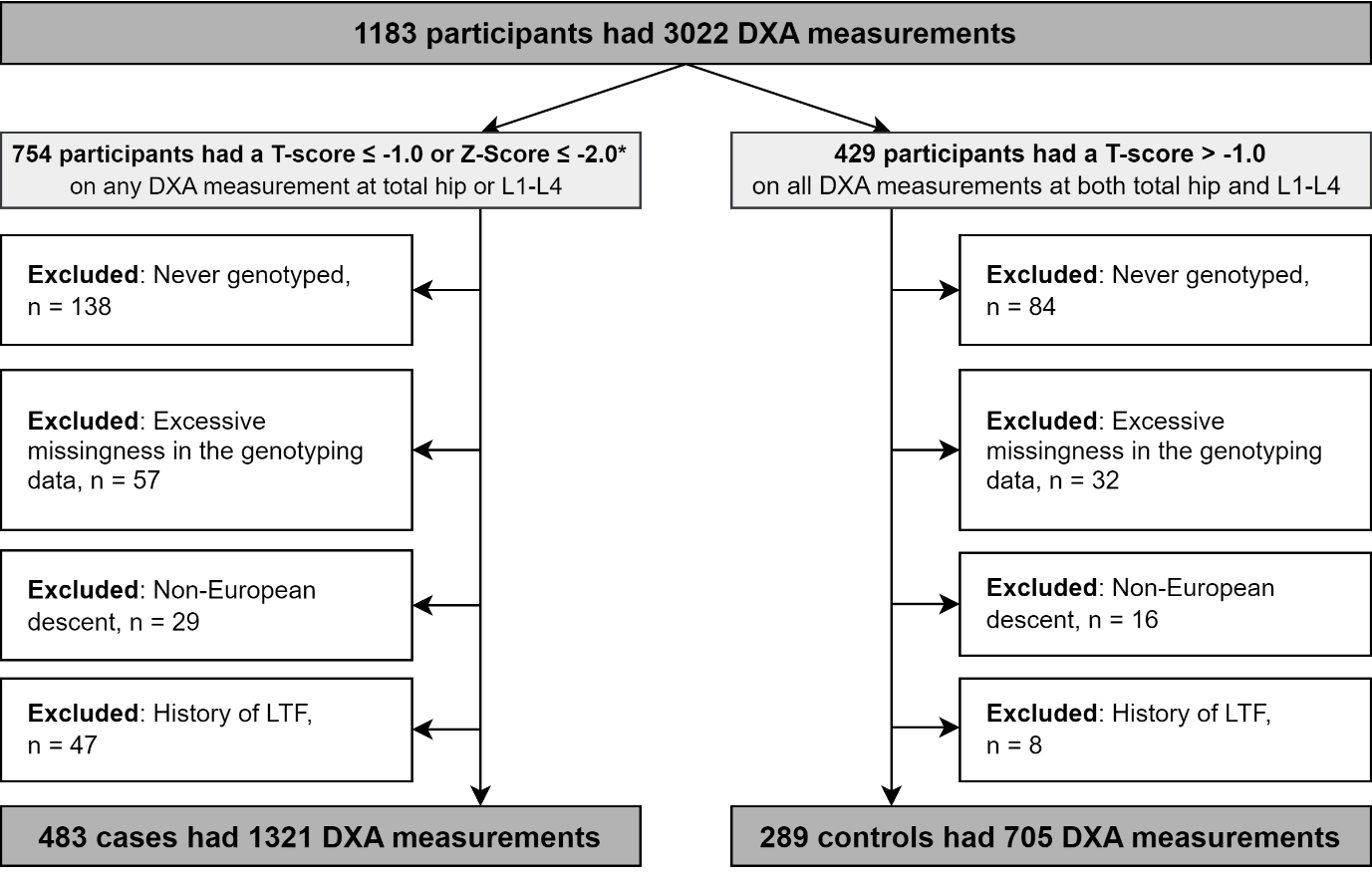
**

***** Z-Scores were considered for premenopausal women and men <50 years at the time of DXA-measurement.

**Abbreviations**: DXA, dual-energy X-ray absorptiometry; L1-L4, lumbar spine segments 1-4; LTF, low trauma fracture.

# Supplementary Results

## Supplementary Table 1: Single Nucleotide Polymorphisms included in the different PRS for Osteoporosis/Osteopenia

**Longevity PRS**

| **rsID** | **Gene** | **effect_allele** | **reference_allele** | **Beta_GWAS** | **SE_GWAS** |
| --- | --- | --- | --- | --- | --- |
| rs7412 | *APOE* | C | T | 0.2452 | 0.0367 |
| rs6859 | *NECTIN2* | G | A | -0.1124 | 0.02 |
| rs429358 | *APOE* | T | C | 0.5098 | 0.0322 |
| rs405509 | *APOE* | G | T | -0.1299 | 0.0199 |

**Abbreviations.** PRS, polygenic risk score.

## Supplementary Table 2: Osteoporosis Odds Ratio (OR) According to gSOS Polygenic Risk Score, Clinical Risk Factors, and TDF and bPI, Univariable and Multivariable Analysis.

|  | **Univariable Analysis** | **Multivariable Analysis, adjusted for quintiles of combined clinical risk factors** | **Multivariable Analysis, adjusted for quintiles of clinical risk factors and cumulative TDF/bPI exposure** |
| --- | --- | --- | --- |
|  | OR (95% CI); p-value | OR (95% CI); p-value | OR (95% CI); p-value |
| gSOS 2^nd^ quintile vs 1^st^ quintile | 2.43 (1.18-5.03); 0.016 | 2.8 (1.26-6.22); 0.012 | 2.53 (1.11-5.75); 0.027 |
| gSOS 3^rd^ quintile vs 1^st^ quintile | 2.74 (1.33-5.65); 0.006 | 2.84 (1.28-6.29); 0.010 | 2.88 (1.27-6.55); 0.011 |
| gSOS 4^th^ quintile vs 1^st^ quintile | 3.44 (1.69-7.03); 0.001 | 2.73 (1.25-5.97); 0.012 | 2.7 (1.2-6.03); 0.016 |
| gSOS 5^th^ quintile vs 1^st^ quintile | 4.76 (2.34-9.67); <0.001 | 4.26 (1.95-9.29); <0.001 | 4.13 (1.86-9.18); <0.001 |
|  | **Univariable Analysis** | **Multivariable Analysis, adjusted for quintiles of TDF/bPI** | **Multivariable Analysis, adjusted for TDF/bPI and gSOS PRS** |
|  | OR (95% CI); p-value | OR (95% CI); p-value | OR (95% CI); p-value |
| Combined clinical risk factors 2^nd^ quintile vs 1^st^ quintile | 1.74 (0.78-3.91); 0.179 | 1.64 (0.72-3.72); 0.238 | 1.72 (0.75-3.96); 0.198 |
| Combined clinical risk factors 3^rd^ quintile vs 1^st^ quintile | 3.38 (1.58-7.24); 0.002 | 3.48 (1.6-7.56); 0.002 | 3.38 (1.54-7.44); 0.002 |
| Combined clinical risk factors 4^th^ quintile vs 1^st^ quintile | 8.03 (3.81-16.93); <0.001 | 7.79 (3.64-16.67); <0.001 | 7.09 (3.26-15.41); <0.001 |
| Combined clinical risk factors 5^th^ quintile vs 1^st^ quintile | 16.87 (7.89-36.07); <0.001 | 15.16 (6.96-33.02); <0.001 | 14.86 (6.7-32.94); <0.001 |
|  | **Univariable Analysis** | **Multivariable Analysis, adjusted for quintiles of combined clinical risk factors** | **Multivariable Analysis, adjusted for quintiles of clinical risk factors and gSOS PRS** |
|  | OR (95% CI); p-value | OR (95% CI); p-value | OR (95% CI); p-value |
| TDF exposure (per 5 years) | 1.84 (1.4-2.43); <0.001 | 1.75 (1.27-2.4); 0.001 | 1.65 (1.2-2.29); 0.002 |
| bPI exposure (per 5 years) | 1.44 (1.18-1.76); <0.001 | 1.12 (0.89-1.41); 0.347 | 1.18 (0.92-1.5); 0.187 |

**Abbreviations.** CI, confidence interval; gSOS, genetically predicted heel quantitative ultrasound speed of sound; bPI, boosted protease inhibitor; TDF, tenofovir disoproxil fumarate; OR, odds ratio; PRS, polygenic risk score.

## Supplementary Table 3: Sensitivity Analysis 1: Osteoporosis Odds Ratio (OR) According to Quintiles of gSOS-PRS using the Same Case Definition as in the Primary Analysis and a Control Definition of T-score > -2.5 at all DXA Scans.

|  |  | **Univariable Analysis**  OR (95% CI); p-value | **Multivariable Analysis**  OR (95% CI); p-value |  |
| --- | --- | --- | --- | --- |
|  |  |  |  |  |
| 1^st^ Quintile of gSOS-PRS |  | 1 (reference) | 1 (reference) |  |
| 2^nd^ Quintile of gSOS-PRS |  | 1.75 (0.91-3.36); 0.093 | 1.63 (0.81-3.26); 0.171 |  |
| 3^rd^ Quintile of gSOS-PRS |  | 2.24 (1.19-4.23); 0.012 | 2.41 (1.23-4.73); 0.010 |  |
| 4^th^ Quintile of gSOS-PRS |  | 2.47 (1.32-4.61); 0.005 | 2.05 (1.05-3.99); 0.035 |  |
| 5^th^ Quintile of gSOS-PRS |  | 2.69 (1.44-5.04); 0.002 | 2.34 (1.2-4.57); 0.013 |  |
| Age (per 10 years) |  | Per 10 years older, 1.31 (1.03-1.66); 0.026 | Per 10 years older, 1.37 (1.05-1.79); 0.020 |  |
| Menopause^1^ |  | 3.51 (1.24-9.95); 0.018 | - |  |
| BMI categories | underweight (BMI <18.5) | 4.07 (1.96-8.46); <0.001 | 3.76 (1.71-8.3); 0.001 |  |
|  | normal (BMI 18.5-24.9) | 1 (reference) | 1 (reference) |  |
|  | overweight (BMI 25-29.9) | 0.32 (0.2-0.52); <0.001 | 0.33 (0.2-0.54); <0.001 |  |
|  | obese (BMI >30) | 0.26 (0.1-0.65); 0.004 | 0.32 (0.12-0.84); 0.020 |  |
| Leisure activity (>20min) >=1/week |  | 0.73 (0.51-1.05); 0.086 | - |  |
| HIV acquisition mode | heterosexual | Male: 1.12 (0.67-1.86); 0.668 | Male: 1.1 (0.62-1.93); 0.747 |  |
|  |  | Female: 1.13 (0.64-2.01); 0.669 | Female: 0.94 (0.5-1.76); 0.837 |  |
|  | MSM | 1 (reference) | 1 (reference) |  |
|  | IDU | Male: 1.7 (0.95-3.04); 0.073 | Male: 1.23 (0.54-2.81); 0.616 |  |
|  |  | Female: 2.59 (1.15-5.83); 0.022 | Female: 1.75 (0.62-4.97); 0.293 |  |
|  | other | 0.37 (0.09-1.61); 0.186 | 0.48 (0.11-2.17); 0.342 |  |
| Smoking | current | 1.16 (0.75-1.78); 0.509 | - |  |
|  | past | 1.11 (0.7-1.77); 0.658 | - |  |
|  | never | 1 (reference) | - |  |
| Alcohol consumption | none/mild | 1 (reference) | - |  |
|  | moderate/heavy | 0.83 (0.57-1.18); 0.297 | - |  |
| Diabetes mellitus |  | 0.48 (0.19-1.24); 0.128 | 0.48 (0.17-1.37); 0.172 |  |
| Dyslipidemia |  | 0.82 (0.57-1.17); 0.269 | - |  |
| Lipid lowering therapy |  | 0.99 (0.6-1.62); 0.968 | - |  |
| Corticotherapy > 3 months |  | 0.58 (0.24-1.39); 0.223 | - |  |
| Hepatitis C seropositivity |  | 1.79 (1.17-2.74); 0.007 | 1.1 (0.56-2.18); 0.773 |  |
| Parent hip fracture |  | 1.79 (1.02-3.15); 0.043 | - |  |
| Tenofovir disoproxil fumarate, per 5 years exposure |  | Per 5 years exposure: 1.72 (1.34-2.19); <0.001 | Per 5 years exposure: 1.64 (1.25-2.15); <0.001 |  |
| Boosted protease inhibitor, median exposure (IQR), years |  | Per 5 years exposure: 1.31 (1.1-1.56); 0.003 | Per 5 years exposure: 1.08 (0.88-1.32); 0.484 |  |
| CD4 nadir (cells/μL) |  | 0.89 (0.78-1.02); 0.093 | 1.06 (0.9-1.23); 0.494 |  |
| CD4 nadir <50 cells/μL |  | 0.95 (0.56-1.62); 0.853 | - |  |
| CD4 (cells/μL) |  | 1 (0.94-1.06); 0.909 | - |  |
| HIV RNA <50 copies/mL (undetectable) |  | 1.11 (0.51-2.44); 0.790 | - |  |
| Maximal HIV RNA (copies/mL) |  | 0.89 (0.74-1.06); 0.195 | - |  |

^1^ Menopause status was only considered for female participants, n = 127.

**Abbreviations.** BMI, body mass index; CI, confidence interval; gSOS, genetically predicted heel quantitative ultrasound speed of sound; OR, odds ratio; PRS, polygenic risk score.

## Supplementary Table 4: Sensitivity Analysis 2: Osteoporosis Odds Ratio (OR) According to Quintiles of gSOS-PRS using a T-Score < -1.0 or Z-score < -2 in premenopausal women or men <50 years as Case Definition, Participants with a T-Score > -1.0 on all DXA Scans being Controls.

|  |  | **Univariable Analysis**  OR (95% CI); p-value | **Multivariable Analysis**  OR (95% CI); p-value |  |
| --- | --- | --- | --- | --- |
|  |  |  |  |  |
| 1^st^ Quintile of gSOS-PRS |  | 1 (reference) | 1 (reference) |  |
| 2^nd^ Quintile of gSOS-PRS |  | 1.25 (0.8-1.96); 0.323 | 1.26 (0.78-2.04); 0.349 |  |
| 3^rd^ Quintile of gSOS-PRS |  | 1.51 (0.96-2.38); 0.072 | 1.57 (0.97-2.55); 0.066 |  |
| 4^th^ Quintile of gSOS-PRS |  | 1.59 (1.01-2.5); 0.045 | 1.36 (0.83-2.22); 0.217 |  |
| 5^th^ Quintile of gSOS-PRS |  | 2.53 (1.56-4.1); <0.001 | 2.3 (1.37-3.88); 0.002 |  |
| Age (per 10 years) |  | Per 10 years older, 1.16 (0.95-1.43); 0.147 | Per 10 years older, 1.21 (0.97-1.51); 0.097 |  |
| Menopause^1^ |  | 4.36 (2.01-9.46); <0.001 | - |  |
| BMI categories | underweight (BMI <18.5) | 12.16 (1.64-90.06); 0.014 | 11.21 (1.48-85.11); 0.019 |  |
|  | normal (BMI 18.5-24.9) | 1 (reference) | 1 (reference) |  |
|  | overweight (BMI 25-29.9) | 0.37 (0.26-0.51); <0.001 | 0.37 (0.26-0.52); <0.001 |  |
|  | obese (BMI >30) | 0.23 (0.14-0.4); <0.001 | 0.27 (0.15-0.48); <0.001 |  |
| Leisure activity (>20min) >=1/week |  | 0.72 (0.54-0.97); 0.028 | 0.7 (0.51-0.96); 0.029 |  |
| HIV acquisition mode | heterosexual | Male: 0.91 (0.61-1.37); 0.660 | Male: 0.94 (0.6-1.47); 0.785 |  |
|  |  | Female: 0.77 (0.49-1.21); 0.253 | Female: 0.62 (0.37-1.03); 0.063 |  |
|  | MSM | 1 (reference) | 1 (reference) |  |
|  | IDU | Male: 1.96 (1.1-3.49); 0.023 | Male: 1.66 (0.78-3.53); 0.187 |  |
|  |  | Female: 3.7 (1.26-10.85); 0.017 | Female: 2.29 (0.68-7.65); 0.180 |  |
|  | other | 0.66 (0.3-1.45); 0.303 | 0.75 (0.33-1.72); 0.499 |  |
| Smoking | current | 1.39 (0.98-1.97); 0.065 | - |  |
|  | past | 1 (0.69-1.44); 0.994 | - |  |
|  | never | 1 (reference) | - |  |
| Alcohol consumption | none/mild | 1 (reference) | - |  |
|  | moderate/heavy | 1.1 (0.82-1.47); 0.524 | - |  |
| Diabetes mellitus |  | 0.5 (0.28-0.91); 0.023 | 0.55 (0.28-1.08); 0.083 |  |
| Dyslipidemia |  | 1.07 (0.8-1.43); 0.667 | - |  |
| Lipid lowering therapy |  | 1.18 (0.79-1.78); 0.421 | - |  |
| Corticotherapy > 3 months |  | 1.1 (0.6-2.02); 0.765 | - |  |
| Hepatitis C seropositivity |  | 1.72 (1.15-2.59); 0.009 | 0.85 (0.48-1.53); 0.597 |  |
| Parent hip fracture |  | 0.98 (0.58-1.65); 0.938 | - |  |
| Tenofovir disoproxil fumarate, per 5 years exposure |  | Per 5 years exposure: 1.34 (1.09-1.64); 0.005 | Per 5 years exposure: 1.26 (1-1.58); 0.046 |  |
| Boosted protease inhibitor, median exposure (IQR), years |  | Per 5 years exposure: 1.27 (1.09-1.49); 0.003 | Per 5 years exposure: 1.07 (0.89-1.28); 0.488 |  |
| CD4 nadir (cells/μL) |  | 0.87 (0.79-0.97); 0.009 | 0.95 (0.85-1.07); 0.419 |  |
| CD4 nadir <50 cells/μL |  | 1.11 (0.72-1.72); 0.632 | - |  |
| CD4 (cells/μL) |  | 0.95 (0.91-1); 0.067 | - |  |
| HIV RNA <50 copies/mL (undetectable) |  | 0.83 (0.44-1.56); 0.558 | - |  |
| Maximal HIV RNA (copies/mL) |  | 0.92 (0.79-1.08); 0.334 | - |  |

^1^ Menopause status was only considered for female participants, n = 127.

**Abbreviations.** BMI, body mass index; CI, confidence interval; gSOS, genetically predicted heel quantitative ultrasound speed of sound; OR, odds ratio; PRS, polygenic risk score.

## Supplementary Table 5: Sensitivity Analysis 3: Osteoporosis Odds Ratio (OR) Including only Injection Drug Use, but not Hepatitis C Seropositivity in the Multivariable Model.

|  |  | **Multivariable Analysis**  OR (95% CI); p-value |  |
| --- | --- | --- | --- |
|  |  |  |  |
| 1^st^ Quintile of gSOS-PRS |  | 1 (reference) |  |
| 2^nd^ Quintile of gSOS-PRS |  | 1.81 (0.80-4.08); 0.154 |  |
| 3^rd^ Quintile of gSOS-PRS |  | 2.46 (1.12-5.39); 0.025 |  |
| 4^th^ Quintile of gSOS-PRS |  | 2.16 (0.99-4.73); 0.054 |  |
| 5^th^ Quintile of gSOS-PRS |  | 3.27 (1.46-7.32); 0.004 |  |
| Age (per 10 years) |  | Per 10 years older, 1.45 (1.05-2.01); 0.023 |  |
| BMI categories | underweight (BMI <18.5) | 19.43 (2.40-157.56); 0.005 |  |
|  | normal (BMI 18.5-24.9) | 1 (reference) |  |
|  | overweight (BMI 25-29.9) | 0.22 (0.12-0.38); <0.001 |  |
|  | obese (BMI >30) | 0.20 (0.07-0.56); 0.002 |  |
| Leisure activity (>20min) >=1/week |  | 0.66 (0.41-1.06); 0.082 |  |
| HIV acquisition mode | heterosexual | Male: 0.81 (0.41-1.59); 0.535 |  |
|  |  | Female: 0.60 (0.28-1.27); 0.182 |  |
|  | MSM | 1 (reference) |  |
|  | IDU | Male: 1.34 (0.56-3.17); 0.512 |  |
|  |  | Female: 2.36 (0.65-8.55); 0.193 |  |
|  | other | 0.38 (0.08-1.87); 0.235 |  |
| Diabetes mellitus |  | 0.42 (0.13-1.41); 0.159 |  |
| Tenofovir disoproxil fumarate, per 5 years exposure |  | Per 5 years exposure: 1.78 (1.27-2.50); 0.001 |  |
| Boosted protease inhibitor, median exposure (IQR), years |  | Per 5 years exposure: 1.12 (0.86-1.46); 0.380 |  |
| CD4 nadir (cells/μL) |  | 1.03 (0.87-1.22); 0.752 |  |

**Abbreviations.** BMI, body mass index; CI, confidence interval; gSOS, genetically predicted heel quantitative ultrasound speed of sound; OR, odds ratio; PRS, polygenic risk score.

## Supplementary Table 6: Sensitivity Analysis 4: Osteoporosis Odds Ratio (OR) Including only Hepatitis C Seropositivity, but not Injection Drug Use in the Multivariable Model.

|  |  | **Multivariable Analysis**  OR (95% CI); p-value |  |
| --- | --- | --- | --- |
|  |  |  |  |
| 1^st^ Quintile of gSOS-PRS |  | 1 (reference) |  |
| 2^nd^ Quintile of gSOS-PRS |  | 1.75 (0.78-3.93); 0.174 |  |
| 3^rd^ Quintile of gSOS-PRS |  | 2.51 (1.16-5.45); 0.020 |  |
| 4^th^ Quintile of gSOS-PRS |  | 2.17 (1.00-4.71); 0.051 |  |
| 5^th^ Quintile of gSOS-PRS |  | 3.43 (1.54-7.63); 0.003 |  |
| Age (per 10 years) |  | Per 10 years older, 1.42 (1.03-1.95); 0.030 |  |
| BMI categories | underweight (BMI <18.5) | 16.55 (2.07-132.01); 0.008 |  |
|  | normal (BMI 18.5-24.9) | 1 (reference) |  |
|  | overweight (BMI 25-29.9) | 0.22 (0.12-0.38); <0.001 |  |
|  | obese (BMI >30) | 0.19 (0.07-0.54); 0.002 |  |
| Leisure activity (>20min) >=1/week) |  | 0.67 (0.42-1.07); 0.096 |  |
| HIV acquisition mode | MSM | 1 (reference) |  |
|  | Heterosexual male | 0.88 (0.49-1.58); 0.666 |  |
|  | Female | 0.74 (0.37-1.47); 0.383 |  |
|  | Other | 0.33 (0.07-1.67); 0.181 |  |
| Hepatitis C seropositivity |  | 1.53 (0.79-2.97); 0.205 |  |
| Diabetes mellitus |  | 0.38 (0.11-1.26); 0.113 |  |
| Tenofovir disoproxil fumarate, per 5 years exposure |  | Per 5 years exposure: 1.78 (1.28-2.49); 0.001 |  |
| Boosted protease inhibitor, median exposure (IQR), years |  | Per 5 years exposure: 1.11 (0.85-1.44); 0.435 |  |
| CD4 nadir (cells/μL) |  | 1.01 (0.85-1.20); 0.893 |  |

**Abbreviations.** BMI, body mass index; CI, confidence interval; gSOS, genetically predicted heel quantitative ultrasound speed of sound; OR, odds ratio; PRS, polygenic risk score.

# Bibliography

1. Durbin R. Efficient haplotype matching and storage using the positional Burrows-Wheeler transform (PBWT). Bioinformatics **2014**; 30:1266–1272.

2. McCarthy S, Das S, Kretzschmar W, et al. A reference panel of 64,976 haplotypes for genotype imputation. Nat Genet **2016**; 48:1279–1283.

3. Loh P-R, Danecek P, Palamara PF, et al. Reference-based phasing using the Haplotype Reference Consortium panel. Nat Genet **2016**; 48:1443–1448.

4. Price AL, Patterson NJ, Plenge RM, Weinblatt ME, Shadick NA, Reich D. Principal components analysis corrects for stratification in genome-wide association studies. Nat Genet **2006**; 38:904–909.

5. The International HapMap 3 Consortium. Integrating common and rare genetic variation in diverse human populations. Nature **2010**; 467:52–58.

6. Manichaikul A, Mychaleckyj JC, Rich SS, Daly K, Sale M, Chen W-M. Robust relationship inference in genome-wide association studies. Bioinformatics **2010**; 26:2867–2873.
